# Differential DNA methylation marks and gene comethylation of COPD in African-Americans with COPD exacerbations

**DOI:** 10.1186/s12931-016-0459-8

**Published:** 2016-11-05

**Authors:** Robert Busch, Weiliang Qiu, Jessica Lasky-Su, Jarrett Morrow, Gerard Criner, Dawn DeMeo

**Affiliations:** 1 Channing Division of Network Medicine, Department of Medicine, Brigham and Women’s Hospital, 181 Longwood Ave, Room 449, Boston, 02111 MA USA; 2 Temple Lung Center, Temple University Health System, Philadelphia, PA USA

**Keywords:** Chronic obstructive pulmonary disease, DNA methylation, Microarray, Weighted gene coexpression network analysis, Smoking

## Abstract

**Background:**

Chronic obstructive pulmonary disease (COPD) is the third-leading cause of death worldwide. Identifying COPD-associated DNA methylation marks in African-Americans may contribute to our understanding of racial disparities in COPD susceptibility. We determined differentially methylated genes and co-methylation network modules associated with COPD in African-Americans recruited during exacerbations of COPD and smoking controls from the Pennsylvania Study of Chronic Obstructive Pulmonary Exacerbations (PA-SCOPE) cohort.

**Methods:**

We assessed DNA methylation from whole blood samples in 362 African-American smokers in the PA-SCOPE cohort using the Illumina Infinium HumanMethylation27 BeadChip Array. Final analysis included 19302 CpG probes annotated to the nearest gene transcript after quality control. We tested methylation associations with COPD case-control status using mixed linear models. Weighted gene comethylation networks were constructed using weighted gene coexpression network analysis (WGCNA) and network modules were analyzed for association with COPD.

**Results:**

There were five differentially methylated CpG probes significantly associated with COPD among African-Americans at an FDR less than 5 %, and seven additional probes that approached significance at an FDR less than 10 %. The top ranked gene association was *MAML1*, which has been shown to affect *NOTCH*-dependent angiogenesis in murine lung. Network modeling yielded the “yellow” and “blue” comethylation modules which were significantly associated with COPD (*p*-value 4 × 10^-10^ and 4 × 10^-9^, respectively). The yellow module was enriched for gene sets related to inflammatory pathways known to be relevant to COPD. The blue module contained the top ranked genes in the concurrent differential methylation analysis (*FXYD1/LGI4*, gene significance *p*-value 1.2 × 10^-26^; *MAML1*, *p*-value 2.0 × 10^-26^; *CD72*, *p*-value 2.1 × 10^-25^; and *LPO*, *p*-value 7.2 × 10^-25^), and was significantly associated with lung development processes in Gene Ontology gene-set enrichment analysis.

**Conclusion:**

We identified 12 differentially methylated CpG sites associated with COPD that mapped to biologically plausible genes. Network module comethylation patterns have identified candidate genes that may be contributing to racial differences in COPD susceptibility and severity. COPD-associated comethylation modules contained genes previously associated with lung disease and inflammation and recapitulated known COPD-associated genes. The genes implicated by differential methylation and WGCNA analysis may provide mechanistic targets contributing to COPD susceptibility, exacerbations, and outcomes among African-Americans.

**Trial registration:**

Trial Registration: NCT00774176, Registry: ClinicalTrials.gov, URL: www.clinicaltrials.gov, Date of Enrollment of First Participant: June 2004, Date Registered: 04 January 2008 (retrospectively registered).

**Electronic supplementary material:**

The online version of this article (doi:10.1186/s12931-016-0459-8) contains supplementary material, which is available to authorized users.

## Background

Chronic obstructive pulmonary disease (COPD) is an incurable lung disease characterized by progressive airflow obstruction involving emphysematous destruction of lung parenchyma and mucus hypersecretion with chronic bronchitis. Over 12 million Americans are affected by COPD, which is the third leading cause of death in the US, [1] and projected to become the third leading cause of death worldwide [2]. Recent data suggest that the prevalence of emphysema, chronic bronchitis, and COPD hospitalizations are increasing among African-Americans (AA), [3–5] and that AA may develop COPD at a younger age than those who racially self-identify as white (WH) [5]. In addition, AA males have one of the highest prevalence rates of smoking (25.5 %) among racial groups in the United States, [6] leading to a predictable growing burden of lung disease in this group. AA individuals present with similar severity of airflow obstruction as WH, despite fewer pack-years of smoking [5]. Once they have developed COPD, AA have lower quality of life scores [7]. Despite these alarming trends, COPD has been understudied in African-Americans.

Race is an important contributor to genetic [8] and epigenetic variability, and recent studies have identified epigenetic association signals that differ between racial groups [9]. Similarly, the results of differential methylation association studies of complex traits in single racial-ancestry cohorts may miss epigenetic risk factors in another racial-ancestry cohort, and may not be generalizable to other racial cohorts at all [10, 11]. Recent methylation studies have shown a subset of methylation signals particular to AA smokers, [9] but to our knowledge investigations of epigenetic associations in AA with COPD have not been previously performed. Understanding the epigenetic associations of smoking and COPD in AA current and former smokers may provide insights into features relevant to COPD-related disparities in AA that may inform treatment within these groups as well as point out disease pathways applicable to all people with COPD.

DNA methylation patterns are determined at multiple time points in the life of an individual, [12] including *in utero* during imprinting, tissue-specific methylation during development, and changes in the methylation of genes in response to major environmental exposures. Differential methylation impacts gene regulation, which may lead to clinically relevant changes in disease-related phenotypes. Modules of genes with correlated comethylation profiles may identify groups of genes under similar regulation that are associated with COPD risk. Prior research has identified differential methylation signals related to tobacco smoke exposure that may influence risk for development of COPD [13–18]. The majority of these studies have focused on WH subjects as the largest proportion of their cohorts. Our investigation focused on the identification of differential methylation sites associated with COPD as well as COPD-associated comethylation modules in an AA cohort (the Pennsylvania Study of Chronic Obstructive Pulmonary Exacerbations, PA-SCOPE), with comparison to a separate WH cohort (the International COPD Genetics Network, ICGN). Our hypothesis was that patterns of DNA methylation in AA would identify differentially methylated genes or comethylation networks relevant to COPD in AA that may not be significantly associated in WH cohorts. A better understanding of the epigenetic factors associated with the features of COPD in AA smokers may provide insights into new diagnostic options, drive the discovery and targeting of therapeutics, and improve primary prevention strategies in this susceptible population.

## Results

**Fig. 1:**
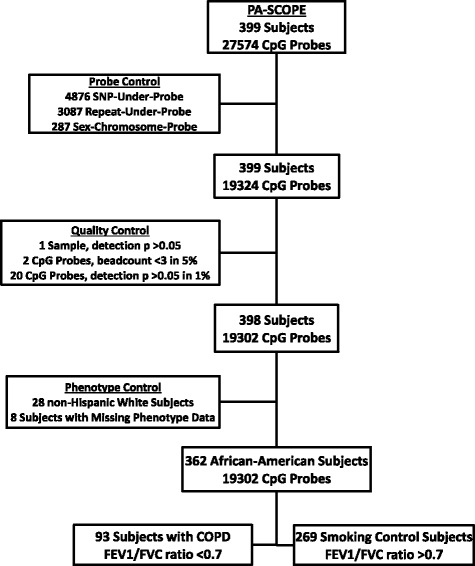
Subject- and Probe-Level Quality Control Chart. Quality control of the PA-SCOPE methylation dataset included Probe-level controls and Subject-level quality controls (see Methods for details). Final analysis included 93 COPD cases and 269 smoking controls. SNP-Under-Probe refers to probes containing a CpG within 5 base pairs upstream or downstream of a known genomic SNP. Repeat-Under-Probe refers to probes that mapped to genomic repeat regions

After quality control, the PA-SCOPE dataset included methylation data on 19302 probes measured in 93 AA subjects with COPD defined by GOLD spirometry criteria (GOLD I-IV), as well as 269 smoking controls. A quality control schematic is provided in Fig. 1. Technical replicates for one male and one female sample plated repeatedly showed over 99 % intra-sample methylation concordance. Baseline statistics for PA-SCOPE cases and controls showed expected differences in metrics used to define COPD severity including forced expiratory volume in 1 s as percent predicted (FEV1), forced vital capacity as percent predicted (FVC), and the ratio of FEV1 to FVC (FEV1/FVC), while pack-year history of smoking (PYH) was similar. Baseline data for PA-SCOPE and ICGN is presented in Tables 1 and 2.

**Table 1:** Baseline statistics among African-Americans in PA-SCOPE

	Cases		Controls	
*N*=	93		269	
Age (years)	61.4	±7.7	48.9	± 6.4
FEV1/FVC Ratio	0.434	± 0.123	0.799	± 0.053
FEV1 percent predicted	36.8	± 15.4	89.9	± 12.5
FVC percent predicted	67.5	± 22.4	90.7	± 12.3
Gender (% male)	50	53.8	195	72.5
Pack-Years of Smoking	42.2	± 22.1	39.2	± 20.5

**Table 2:** Baseline statistics among whites in ICGN

	Cases		Controls	
*N*=	678		427	
Age (years)	59.2	± 7.4	54.9	± 9.0
FEV1/FVC Ratio	0.412	± 0.137	0.747	± 0.052
FEV1 percent predicted	41.3	± 16.8	99.6	± 14.4
FVC percent predicted	73.2	± 20.0	106.3	± 15.7
Gender (% male)	389	57.4	216	0.506
Pack Years of Smoking	49.7	± 26.0	29.4	± 20.7

Data is presented as count with proportion in parentheses or mean with standard deviation

### Differential DNA Methylation Analysis

We used linear mixed models to identify COPD-associated CpG sites, using the method of Benjamini and Hochberg to control type I error. Five differentially methylated CpG sites were associated with COPD (Table 3) at FDR less than 5 %. All of these five CpG sites exhibited relative hypomethylation in association with the presence of COPD. The mean difference in percent methylation between cases and controls among the top five associated sites ranged from 5.3 to 9.6 %. The top differentially methylated CpG site was cg16361890 (unadjusted *p*-value 8.188 × 10^-8^, percent methylation change -7.8 %), which mapped to the *MAML1* gene. Gene annotation for the remaining four differentially methylated CpG sites associated with COPD included *RBFOX2*, *CD72*, *GRASP*, and *SH3TC1*.

**Table 3:** Differentially Methylated CpG Probes Associated with COPD Among African- Americans in PA-SCOPE

Chromosome	CpG Probe	Nearest Gene	Mean Difference in Percent Methylation	*p*-value	FDR Adjusted *p*-value
5	cg16361890	*MAML1*	-7.8	8.19E-08	0.001
22	cg00615377	*RBFOX2*	-5.3	1.20E-07	0.001
9	cg12971694	*CD72*	-9.0	2.05E-06	0.013
12	cg22566906	*GRASP*	-7.8	7.27E-06	0.029
4	cg02635407	*SH3TC1*	-9.6	7.47E-06	0.029
11	cg25634666	*FOLR3*	-10.8	3.23E-05	0.080
10	cg18390025	*ELOVL3*	-8.4	3.40E-05	0.080
5	cg10257049	*FAXDC2*	-7.5	3.58E-05	0.080
19	cg27461196	*FXYD1/LGI4*	-11.9	4.27E-05	0.080
6	cg00333528	*GABRR1*	-8.0	4.43E-05	0.080
21	cg17356733	*IFNGR2*	-11.9	4.56E-05	0.080
17	cg24489015	*LPO*	-10.8	5.82E-05	0.094

Data are presented in decreasing order of significance. Mean difference in percent methylation represents the mean difference in percent methylation between cases and controls; *p*-values were adjusted for multiple comparisons using the method of Benjamini and Hochberg

**Fig. 2:**
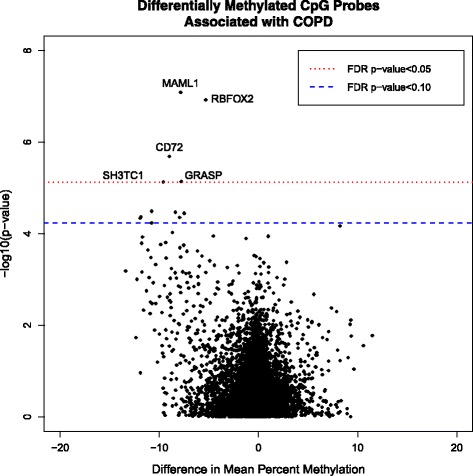
Differentially Methylated CpG Probes Associated with COPD. Differential methylation analysis revealed 12 CpG sites in 12 genes significantly associated with COPD with an FDR-corrected *p*-value less than 0.10. Difference in mean percent methylation represents the difference in mean methylation between COPD cases and smoking controls. The y-axis represents the negative log of the association *p*-value from linear mixed models adjusted for age, gender, pack years of smoking, assay batch, and cell type. The name of the nearest gene is included with each of the top five CpG results

**Fig. 3:**
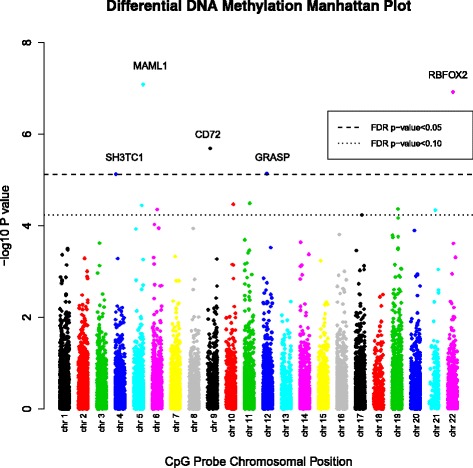
Manhattan Plot of Differential Methylation Analysis Results. Differential methylation analysis results presented by chromosomal location (x-axis). The y-axis represents the negative log of the association *p*-value from linear mixed models adjusted for age, gender, pack years of smoking, assay batch, and cell type. The name of the nearest gene is included with each of the top five CpG results

Seven additional differentially methylated CpG sites were associated with COPD at FDR of less than 10 %, (Table 3). These seven additional CpG sites (annotated to *FOLR3*, *ELOVL3*, *FAXDC2*, *FXYD1*/LGI4, *GABRR1*, *IFNGR2*, and *LPO*) also exhibited relative hypomethylation in association with COPD. The mean difference in methylation between cases and controls ranged from 7.5 to 11.9 %. These differential methylation results are presented in a volcano plot (Fig. 2) and a Manhattan Plot (Fig. 3).

### Comparison to WH Dataset

We next examined the COPD-associated differential methylation sites discovered in the PA-SCOPE AA cohort in the ICGN WH cohort methylation data [19]. We qualitatively compared the magnitude of the difference of COPD-association test-statistic between AA and WH at each differentially methylated CpG site (PA-SCOPE test-statistic minus ICGN test-statistic) from the mixed linear model output (Additional file 1: Table S1 and Figure S1). We posited that a large difference in test statistic at a given CpG site between the PA-SCOPE and ICGN studies could indicate a difference in differential methylation between AA and WH related to COPD case-control status. This difference metric was found to have a normal distribution (mean = -0.316, sd = 1.515) across the ~19000 probes remaining after data cleaning. Seven of the 12 differentially methylated genes identified at FDR less than 10 % in the primary PA-SCOPE analysis were found to have test-statistic-difference values in the lower 2.5 percentile tail of this distribution, supporting the presence of statistically significant differential methylation at these sites in the presence of COPD in AA but not in WH.

### Weighted gene comethylation network analysis

**Fig. 4:**
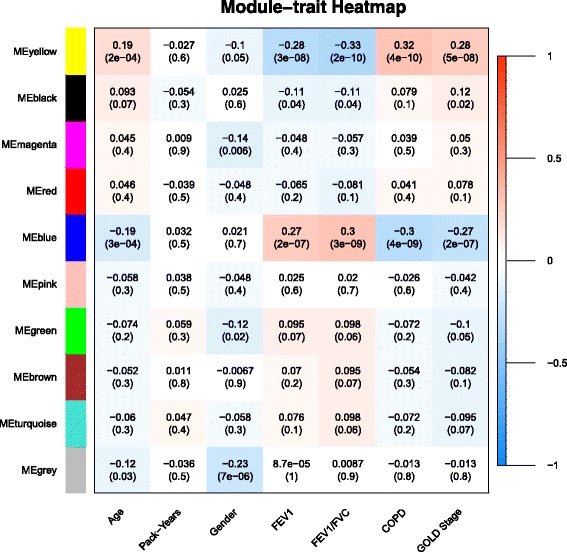
WCGNA Module Trait Relationship Heatmap. Heatmap showing comethylation module correlation with phenotypic trait and associated *p*-value for these correlations within PA-SCOPE. Positive or negative correlation magnitude with COPD is presented with *p*-value for the correlation with COPD in parenthesis. The yellow and blue modules were both significantly associated with COPD affection status, labeled “COPD”

Weighted Gene Coexpression Network Analysis software (WGCNA) was used to create a scale-free comethylation network (see Additional file 1: Figure S2) [20, 21]. The resultant network contained ten modules (see Fig. 4), of which two were significantly correlated with COPD case-control status (labeled “blue” and “yellow” modules). The blue module contained 5009 probes (*p*-value 4 × 10^-9^ for module association with COPD status), and the yellow module contained 1698 probes (*p*-value 4 × 10^-10^ for module association with COPD status). The association of the blue and yellow module eigengenes remained statistically significant (*p*-value 1.6 × 10^-4^ for blue module, 5.5 × 10^-5^ for yellow module) in a logistic regression model of COPD affection status after controlling for age, gender, and pack-years of smoking history.

We investigated the two COPD-associated network modules for genes previously related to COPD. This investigation included genes from the differential methylation analysis results and genes found to be associated with COPD and lung function measurements in genome-wide association studies (GWAS) (see Table 4). CpG sites marking 11 of the 12 top differential methylation sites found in our differential methylation analysis (*GRASP*, *FXYD1/LGI4*, *MAML1*, *FOLR3*, *CD72*, *LPO*, *GABRR1*, *SH3TC1*, *RBFOX2*, *IFNGR2*, *ELOVL3*) were part of the blue module. CpG sites marking the COPD- and lung function-associated genes *NOTCH4*, *SERPINA1*, *FAM13A*, *TNS1*, *PPT2*, *CHRNA5*, *PPAP2B*, *CHRNA3*, *RARB*, *CHRNA4*, *ARMC2*, *CCDC38*, *MECOM*, *ADAMTS19*, *HHIP*, and *ZKSCAN3/ZSCAN31* were also all found within the blue module. We limited both the blue and yellow modules to those genes with a stringent module membership (kME value) cutoff of 0.85 for further analysis, [21] yielding a gene set of 317 members and 151 members, respectively.

**Table 4:** COPD-related Genes from WGCNA blue comethylation module

CpG site	Nearest Gene	Gene Significance to COPD	Gene Significance *p*-value	Module Membership to Blue Module	Module Membership *p*-value
cg22566906	GRASP	-0.52	7.06E-27	0.76	8.62E-71
cg27461196	FXYD1/LGI4	-0.52	1.19E-26	0.76	7.10E-70
cg16361890	MAML1	-0.52	1.98E-26	0.83	1.28E-93
cg14700707	NOTCH4	-0.52	2.61E-26	0.72	1.52E-59
cg25634666	FOLR3	-0.51	1.82E-25	0.78	3.50E-76
cg12971694	CD72	-0.51	2.11E-25	0.84	7.77E-99
cg24489015	LPO	-0.50	7.21E-25	0.79	2.57E-78
cg00333528	GABRR1	-0.50	7.29E-25	0.83	5.25E-94
cg02635407	SH3TC1	-0.50	7.93E-25	0.85	1.02E-102
cg00615377	RBFOX2	-0.49	1.25E-23	0.87	1.20E-111
cg11394785	MAML1	-0.48	5.73E-23	0.78	2.39E-75
cg17356733	IFNGR2	-0.48	1.79E-22	0.83	6.93E-95
cg18390025	ELOVL3	-0.48	1.83E-22	0.84	4.19E-97
cg02181506	SERPINA1	-0.45	8.76E-20	0.74	7.93E-65
cg04536922	FAM13A	-0.45	1.62E-19	0.87	1.49E-114
cg00431050	ELOVL3	-0.39	1.57E-14	0.69	3.83E-52
cg18328334	TNS1	-0.37	2.87E-13	0.54	4.75E-29
cg04845063	FAM13A	-0.36	1.38E-12	0.80	1.97E-84
cg12585943	PPT2	-0.29	2.72E-08	0.59	5.39E-35
cg22563815	CHRNA5	-0.29	2.84E-08	0.52	1.72E-26
cg16505550	PPAP2B	-0.23	7.46E-06	0.38	5.96E-14
cg04603031	CHRNA3	-0.22	2.28E-05	0.66	7.85E-47
cg27486427	RARB	-0.22	3.08E-05	0.90	2.67E-130
cg00318573	CHRNA4	-0.19	1.77E-04	0.58	9.87E-35
cg03874127	ARMC2	-0.18	6.61E-04	0.57	2.36E-33
cg25229172	CCDC38	-0.17	8.09E-04	0.43	8.49E-18
cg19848683	MECOM	-0.14	6.09E-03	0.75	5.35E-67
cg13701109	ADAMTS19	-0.14	7.38E-03	0.71	1.57E-57
cg06334284	PPAP2B	-0.13	1.11E-02	0.82	7.50E-92
cg14228238	MECOM	-0.12	2.55E-02	0.60	1.41E-37
cg14580567	HHIP	-0.04	4.24E-01	0.61	3.32E-39
cg21750589	ZKSCAN3/ZSCAN31	-0.04	4.95E-01	0.26	4.78E-07

Summary of “gene significance to phenotype” values and “blue module membership” values of genes previously associated with COPD and lung function through GWAS, as well as differentially methylated sites discovered in the differential methylation analysis

Gene set enrichment analyses of the limited modules were performed using GO Biological Processes ontology, KEGG, and Reactome pathways. The limited blue module of 317 genes was enriched for developmental gene sets, with statistically significant enrichment found in GO pathways such as anatomical structure development (GO:0048856; adjusted *p*-value 3.6 × 10^-10^), branching morphogenesis of an epithelial tube (GO:0048754; adjusted *p*-value1.5 × 10^-4^), lung morphogenesis (GO:0060425; adjusted *p*-value 0.01), lung lobe morphogenesis (GO:0060425; adjusted *p*-value 0.0246), lung development (GO:0030324; adjusted *p*-value), and lung lobe development (GO:0060428; *p*-value 1.38 × 10^-3^). The limited yellow module of 151 genes was enriched for immune/inflammatory gene sets, such as response to other organism (GO:0051707; adjusted *p*-value 8.3 × 10^-4^), immune response (GO:0006955; adjusted *p*-value 7.3 × 10^-3^), defense response to bacterium (GO:0071219; adjusted *p*-value 4.2 × 10^-3^), and chemotaxis of natural killer cells, neutrophils, and eosinophils (GO:0035747, 0030593, 0048245, respectively; adjusted *p*-values 3.5 × 10^-2^ to 7.2 × 10^-3^).

## Discussion

Within the PASCOPE AA cohort, we identified 5 differentially methylated CpG sites significantly associated with COPD using an FDR of 5 %, and 7 additional associations that approached significance using an FDR of 10 %. We used WGCNA to identify comethylation modules associated with COPD that were enriched for genes related to lung development and immune response and contained biologically relevant genes associated with COPD and lung function. Differentially methylated CpG sites associated with COPD mapped to genes that were biologically plausible candidates for COPD pathogenesis. Notable functions among these genes included *NOTCH4*-dependent lung angiogenesis, alveolar macrophage response pathways, and airway defense mechanisms targeting bacteria, as detailed below. Our results included genes and CpGs implicated in previous studies of obstructive lung disease and exacerbations, including *GRASP* and *IFNGR2* (previous genetic associations with asthma) as well as *FYXD1* (differential methylation associated with response to systemic steroids and COPD) [15, 22]. Only 1 of these 12 differentially methylated CpG sites (cg27461196, mapped to *LGI4*/*FXYD1*) was statistically significantly associated with COPD at an FDR of 5 % in an independent, larger WH COPD methylation dataset cleaned and processed in a comparable way. In addition, in a qualitative comparison of the difference in differential methylation of CpG sites between AA and WH, many of our results were statistically significant for differential hypomethylation only in AA.

The majority of our significantly associated (FDR less than 10 %) differential methylation CpG sites are located in genes that are biologically plausible genes for lung disease that may affect the pulmonary, immune, and vascular biology of COPD based on previously published data. Additionally, many of these genes are expressed in either lung (*MAML1*, *RBFOX2*, *GRASP*, *FAXDC2*, *FXYD1/LGI4*, *IFNGR2*) or whole blood (*IFNGR2*) based on GTEx data showing a median reads per kilobase of transcript per million reads (RPKM) >10 in these tissues, [23] providing further support for their potential effects on lung pathology and disease.

Two of the disease-associated genes were associated with COPD in prior studies. Folate Receptor Gamma (*FOLR3*) was found to be 15- to 20-fold upregulated during stable COPD and acute exacerbations of COPD in previous studies, [24] although the mechanistic and functional implications of this upregulation are unclear. Differential methylation of Phospholemman (*FXYD1*) was shown to be associated with COPD in the ICGN cohort by Qiu et al, [15] and this gene was also previously found to be differentially methylated in response to systemic steroid use in COPD [22]. Both of these genes are notably related to acute exacerbations of COPD as well as a preferred treatment modality (systemic steroids) for acute exacerbations. The PASCOPE study recruited subjects who were hospitalized for acute exacerbations of COPD, and blood draws for DNA methylation analysis were performed during the inpatient hospitalization. Because of the timing of our sampling, the methylation pattern of these genes may be related to a confounder such as acute exacerbations of COPD, systemic steroid use, recent smoking, or to a subset of subjects in our dataset with a phenotype of frequent exacerbations, although this could not be directly assessed based on our data.

Five CpG sites were annotated to genes related to pulmonary and airway physiology. Lactoperoxidase (*LPO*) is secreted by submucosal glands in human bronchi and plays a role in human airway host defense against bacteria [25]. Gamma-aminobutyric acid Receptor1 (*GABRR1*) has been shown to affect alveolar fluid homeostasis in alveolar epithelial type II cells [26]. Upregulated gene expression of Very Long Chain Fatty Acid Elongase3 (*ELOVL3*) has been proposed to contribute to dysregulated lipid droplet formation in pulmonary surfactant in response to particulate exposure [27]. Rare missense mutations of GRP1-Associated Scaffold Protein (*GRASP*) were previously associated with asthma in a Latino cohort [28]. The function of SH3 Domain and Tetratricopeptide Repeats 1 (*SH3TC1*) has not been adequately described in the lung; however, it is implicated in networks related to bronchial airway epithelial cells and cigarette smoking [29].

An additional three CpG sites were annotated to genes related to immune response and steroid synthesis. Cluster of Differentiation 72 (*CD72*) is a CD5 co-ligand involved in hypersensitivity reactions and sarcoidosis, highly expressed in pulmonary alveolar macrophages [30]. Fatty Acid Hydroxylase Domain Containing2 (*FAXDC2*) is implicated in “steroid biosynthesis” through KEGG pathways [31]. Interferon Gamma Receptor 2 (*IFNGR2*) plays a role in activation of macrophages and regulation of Th1 response to intracellular pathogens, with genetic variants previously associated with atopic asthma [32] and pulmonary tuberculosis.

The final two CpG sites were related to cardiovascular processes. Mastermind-Like1 protein (*MAML1*) effects angiogenesis during organ development through NOTCH-dependent signaling in murine lung [33]. RNA-binding Protein Fox-1 Homolog 2 (*RBFOX2*) is a splicing regulator implicated in differentiation of myofibroblasts to skeletal muscle, and diminished expression previously associated with pressure-overload-mediated progression of dilated cardiomyopathy/heart failure; [34] potential impact on airway smooth muscle has not been described.

We present data showing that many of our top COPD-associated CpG sites are located in the lower tail of a histogram of the difference in test-statistic between CpG sites in the African-American PA-SCOPE dataset and the white ICGN dataset (see Additional file 1: Figure S1) using similar model parameters and adjustment for covariates. This finding may represent qualitative evidence that these sites are more differentially methylated in African-Americans compared to whites, although this conclusion must be seen as hypothesis-generating only without a separate properly controlled and matched study design that would be free of confounding by technical artifacts related to batch. Boxplots of the unadjusted absolute methylation at these sites in both PA-SCOPE and ICGN (see Additional file 1: Figure S3) reveal that the methylation difference between cases and controls is consistent with hypomethylation in COPD cases among both AA and WH, however only among the AA subjects is the difference statistically significant. The relative differential hypomethylation of these CpGs among AA subjects compared to WH subjects could be explained by several scenarios. The most mechanistically attractive possibility is that these CpG sites represent differential methylation events in response to gene-environment interactions experienced preferentially by African-Americans. The second mechanistic possibility is that these CpG sites represent blood methylation quantitative trait loci (mQTL) that are influenced by the genetic architecture specific to the population substructure [35] of African-Americans. In both of the preceding scenarios, the differential methylation could in turn impact damage and airflow obstruction through changes in gene expression and protein production, which could present unique targets for intervention. Finally, the differential methylation may simply be a marker of a confounder between methylation state and COPD, tagging a prior or recent exposure (such as smoking) that directly contributed to both disease and CpG methylation through distinct mechanisms. Blood draws in the PA-SCOPE COPD case subjects occurred during inpatient hospitalizations for acute COPD exacerbations, while blood draws for non-COPD control subjects occurred during study-related office visits; this could lead to potential confounders of our COPD associations including COPD exacerbation, inpatient medication use including corticosteroids, exacerbation triggers such as viral or bacterial infections, or other unmeasured variables.

WGCNA identifies modules of comethylated genes starting from the level of thousands of CpG probes and correlates these modules to phenotypic variables. The network creation and module-building processes of WGCNA are informed purely by gene methylation levels, without consideration of case-control status for COPD. Individual genes within the module can then be related to the module eigengenes by measures of module membership and gene significance to the module. This technique identifies driver genes for the module that may help identify biologically meaningful pathways. In our dataset the yellow and blue modules showed significant association with COPD. Yellow module measures of gene significance were predominantly positive (indicating positive correlation of module comethylation in association with COPD) while the blue module contained primarily negative measures of gene significance (indicating negative correlation of module comethylation in association with COPD).

Further investigation of the blue module showed a network with biological significance for obstructive lung disease. The module was statistically enriched for pathways related to lung development, and also contained multiple genes previously associated with COPD and lung function. *SERPINA1* is the gene responsible for alpha-1-antitrypsin deficiency, [36] a known genetic cause of COPD, and this gene was found to be highly significant in the blue module. The blue module also contained multiple genes previously associated with COPD or lung function measurements through GWAS. WGCNA modules are composed of genes with similar methylation states, which could give insight into processes of coregulation between these genes. While the *SEPRINA1* mutations known to cause alpha-1 antitrypsin deficiency are uncommon in AA, one could hypothesize from this data that coregulation of the *SERPINA1* gene through DNA methylation (and other genes related to lung development in the blue module) could contribute to COPD susceptibility in a disease module framework. However, this hypothesis would require further study with larger datasets including additional modalities such as gene expression. Many of the CpG sites found in the differential methylation analysis were also found in the blue module with high measures of module membership (indicating importance of the gene to the module) and high measures of gene significance to COPD. The recapitulation of these CpG sites in the same module as previously known COPD- and lung-function-related genes adds validation to our differential methylation results.

The yellow module, by comparison, contained genes enriched for immune response pathways. Chronic inflammation in response to airway damage from cigarette smoking as well as external pathogens are recognized as integral parts of the pathogenesis of COPD and exacerbations [37–39]. Enrichment for the chemotaxis of effector cells that are known to play a role in COPD pathogenesis (neutrophils, [40] eosinophils, [41, 42] and natural killer cells [43]) were found using yellow module genes with high module membership values. The PA-SCOPE population was ascertained using subjects with disease exacerbations, so this population may have been enriched for signals associated with acute inflammation and immune response [44, 45].

The PA-SCOPE dataset was a retrospective case-control study and so no direct causation can be inferred from results, only associations of CpG sites with disease. DNA methylation in response to smoking is a dynamic process, and effects may be time-dependent; longitudinal profiling of methylomes and phenotypes is needed [16, 46]. Our data did not contain information on the duration of COPD in our subjects, so we could not assess whether this might impact on our results. COPD is often underdiagnosed or diagnosed at more severe stages of disease, [47, 48] however, so duration of COPD could potentially be unreliable in statistical models comparing COPD cases and controls. While our data did contain information related to spirometric severity of COPD, we were underpowered to detect significant differential DNA methylation site associations with COPD severity due to sample size. The methylation dataset for PA-SCOPE did not contain data related to current smoking or time since quitting smoking, and we could not assess the effects that these important variables might have on our differential methylation results in association with COPD. Multiple studies have shown that smoking history affects DNA methylation, and a recent study showed that a subset of these DNA methylation effects are dynamic in a time-dependent fashion after smoking cessation, [46] however our data did not allow us to control for smoking cessation or time since quitting. Data on chronic or inpatient medication use was also not available, which limits our ability to control for these potential confounders. Longitudinal data was not available in PA-SCOPE, so further conclusions integrating clinical stability, clinical progression, or other lung function trajectories [49] associated with CpG sites cannot be made using these data. Without paired gene expression data, it is unclear what effect these differentially methylated sites have on expression of the associated gene products. While both PA-SCOPE and ICGN were studies of COPD subjects and smoking controls, differences in ascertainment of the datasets may influence the conclusions. Notably, the PA-SCOPE dataset compared AA subjects recruited during inpatient COPD exacerbations with controls without known lung disease or recent respiratory illness. The ICGN dataset compared WH subjects with COPD (with no selection criteria related to COPD exacerbations) with control family members. Because of this difference, our differential methylation site associations with COPD could be confounded by potential methylation effects related to COPD exacerbations. Similarly, the comparison of test-statistic differences between ICGN and PA-SCOPE could be influenced by factors other than racial differences in differential methylation related to COPD. Race was determined by self-report and no genetic testing of ancestry or admixture was performed, thus individuals of mixed genetic ancestry who self-identified as African-American may be included in our analyses and these data may be a source of residual confounding. Batch effects between the PA-SCOPE and ICGN assays, differences in ascertainment and study design related to the timing of COPD exacerbations, and baseline differences in the two populations other than racial make-up could also account for the differences in statistical association among these populations, so we present these data points as qualitative and hypothesis-generating for further investigations.

The Illumina Infinium HumanMethylation27 BeadChip Array interrogates only a subset of CpG sites in the human epigenome, and additional unmeasured sites may be differentially methylated in association with COPD. Specifically, the HumanMethylation27 BeadChip’s design focused on CpG sites within transcription start sites of over 14,000 genes and additional coverage of around 200 cancer-related genes [50]. Additional information on genes not represented on the array as well as additional CpGs in promoter regions, enhancer regions, or the gene body might yield additional associations with COPD and would be an area for further investigations. This study focused on DNA methylation; however other epigenetic changes such as histone acetylation and chromatin modification could impact gene regulation and have relevant associations with COPD; these other modalities were not assayed in our study.

We assayed whole blood for DNA methylation signals associated with COPD, but not lung tissue samples. Prior studies have shown associations between smoking and DNA methylation in whole blood [9, 17, 46]. DNA methylation of lung tissue could potentially capture information related to additional airborne environmental exposures relevant to COPD, which might not be present in DNA methylation from peripheral blood alone. While lung tissue DNA methylation could provide additional insight into disease mechanisms, the additional risks and costs of obtaining lung tissue are not trivial, and human lung tissue itself is a heterogeneous mixture of cell types [51]. However, some whole blood CpG sites may recapitulate DNA methylation signals related to lung exposures [52] or lung disease, [53] and we examined our data in this context. While total pack-years of smoking was a covariate within our models, additional unmeasured variables related to environmental exposure may impact the findings. One could hypothesize that disease mechanisms related to organ development, systemic inflammation, immune response, and protease activity might be best represented in whole blood compared to lung tissue, and our results may reflect this. Additional studies including contemporaneous collections of whole blood and lung tissue would be needed to gain additional insight into these relationships. We present statistically significant differentially methylated CpG associations with COPD with strict multiple testing corrections, however these results need replication in separate datasets. Future studies including large populations of both AA and WH would be needed to further validate both the differential methylation results as well as the race-specificity of our results. The recapitulation of many of our differentially methylated genes in network modules strongly associated with COPD provides some biological validation of the importance of these sites to COPD using a different analytic approach.

## Conclusion

In conclusion, we performed differential methylation analysis in African-American subjects and identified 12 CpG sites statistically significantly associated with COPD at an FDR less than 10 %, of which seven are not statistically significant in a WH cohort study of COPD. We also performed weighted gene comethylation network analysis and identified two comethylation modules associated with COPD in AA, one of which included multiple genes related to obstructive lung disease and COPD. This module was enriched for lung-specific gene sets and our results add to insights into molecular mechanisms that may contribute to lung disease disparities in African-Americans. Molecular mechanisms for COPD-related outcomes in AA smokers have not been adequately investigated. While further research is needed to understand the biological consequences of differential methylation of the genes we identified, they represent promising genes for mechanistic investigations of COPD in AA and for the consideration of epigenetic contributions to racial disparity in COPD susceptibility and severity.

## Methods

### Subjects and data collection

Researchers at Temple University and the Pennsylvania Department of Public Health designed the Pennsylvania Study of Chronic Obstructive Pulmonary Exacerbations (PA-SCOPE, ClinicalTrials.gov Identifier: NCT00774176) as a collaborative observational study to identify demographic and genetic factors that contributed to COPD exacerbations among AA smokers with COPD in urban and rural Pennsylvania. Subject recruitment and data gathering occurred between June 2004 and May 2008. Additional details of the PA-SCOPE study design can be found in Additional file 1. Subjects in the PA-SCOPE methylation dataset were selected from the PA-SCOPE AA study population. PA-SCOPE data types for each of 371 AA subjects in the methylation cohort consisted of questionnaire data, spirometry and pulmonary function tests, and DNA extracted from whole blood samples. All subjects in the methylation dataset analyzed were male and female AA smokers (>20 pack-year history) ages 40-80 years of age. Exclusion criteria for the PA-SCOPE study included <20 pack-years of smoking history, life expectancy <6 months due to any cause, alpha-1 antitrypsin deficiency, or a previous diagnosis of pulmonary fibrosis, bronchiectasis, mediastinal mass, or a pulmonary mass. Subjects with asthma by prior history, lack of a significant smoking history, and or evidence of significant spirometric reversibility with bronchodilator (FEV_1_ increase >15 %) were excluded. COPD cases were subject to additional inclusion criteria including inpatient hospitalization for an acute exacerbation of COPD at the time of study entry and a spirometric diagnosis of COPD without significant bronchodilator response. Acute exacerbations of COPD were defined using criteria of worsening dyspnea, increase sputum volume and sputum purulence [54]. Control subjects were assessed at a routine study-related outpatient visit and met inclusion criteria for smoking history and age, but were excluded if they met spirometric criteria for COPD or had a history of a COPD diagnosis. Exclusion criteria in both cases and controls did not include assessment of other smoking-related comorbidities such as coronary artery disease, cerebrovascular disease, non-pulmonary and non-mediastinal malignancies, and diabetes. Race was determined by self-report. Blood draws for COPD cases were performed during inpatient hospitalizations for COPD exacerbations, while blood draws for controls were acquired during the routine study-related office visit. Spirometry of subjects with COPD was performed within 4 to 6 weeks after their inpatient hospitalizations. Participants provided written consent to participate in this study, and the study was approved by the institutional review boards at all participating institutions (Partners IRB: 2005P000453/BWH).

We assessed DNA samples from 371 COPD cases (defined by FEV1/FVC ≤ 0.7 and FEV1 ≤ 80 % predicted) and smoking controls for genome wide differential methylation using the Illumina (San Diego, CA) Infinium HumanMethylation27 BeadChip (Illumina27K). The Illumina27K array assays 27,758 CpG dinucleotides [50] for quantitative measurements of DNA methylation, covering over 14,000 genes. We included only DNA samples that had undergone ≤ 2 freeze-thaw cycles prior to performing the Illumina27K microarray assay. We performed the assay using the manufacturer’s suggested protocol, including standard controls for bisulfite conversion, amplification, hybridization, and extension. We report the percent methylation values ranging from 0 to 100 % (corresponding to beta value of 0 to 1), calculated as the ratio of the fluorescent intensity of the methylated bead type (meth) to the combined locus intensity of methylated and unmethylated bead types (meth + unmeth) plus an offset (beta = meth/(meth + unmeth + 100)). The log2-ratio of the methylated to unmethylated intensities (M-value) was used for association testing [55]. The absolute difference in percent methylation between cases and controls was used to quantify the effect size.

### Quality control

The R programming language [56] and the BioConductor [57] suite of software (packages including methylumi, [58] GenomicRanges, [59] and wateRmelon [60]) were used for data annotation, probe quality control and pruning, and subject-level quality control. The Illumina27K probes were annotated to their nearest gene using hg19 coordinates. To eliminate false associations due to SNPs underlying probe regions, [61] probes containing a CpG overlying a SNP or within 5 base pairs upstream or downstream of a known genomic SNP were eliminated. Probes underlying genomic repeat regions have also been cited as a source of error using Illumina microarrays, [62] and were eliminated. Probes interrogating the sex chromosomes were also eliminated. Subject level quality control eliminated one sample having 1 % of sites with a detection *p*-value greater than 0.05. CpG sites with a beadcount of less than 3 in greater than 5 % of samples, and sites having greater than 1 % of samples with a detection *p*-value greater than 0.05 were also removed; 8 subjects’ demographic data were incomplete and were removed for the final analysis. Technical replicates for one male and one female were included to assess for within-subject correlation across arrays.

### Cell type deconvolution

Differential methylation signals arising primarily from cell type composition of whole blood can bias methylation analysis. Both smoking and COPD are associated with inflammation, and differences in methylation signals between cases and controls based purely on immune cell type proportion in whole blood could confound association analysis results. To control for this bias, we performed cell type deconvolution using the method and software provided by Houseman et al, [63] which uses the principal components of the methylation signatures of whole blood cellular components projected onto a reference sample to create regression covariates to facilitate adjustment for cell type heterogeneity between samples.

### Differential DNA methylation analysis

Differential methylation analysis was performed on 362 samples after quality control using the limma package [64]. For the COPD analysis, associations between differentially methylated probes and COPD case-control status were modeled in a logistic mixed model controlling for age, gender, pack-years of smoking, batch number, and cell type deconvolution (principal component-based covariates accounting for natural killer cells, CD8+ T-cells, CD4+ T-cells, B-cells, and monocytes). Family-wise type I error was controlled using the method of Benjamini-Hochberg to achieve a genome-wide false discovery rate (FDR) threshold of significance of less than 5 %. Additionally, we examined the results that approached statistical significance at less than FDR 10 % level for assessment of additional biologically-plausible targets. The absolute difference in the mean percent methylation value was used to quantify the magnitude and direction of effect for differentially methylated CpG sites; positive values of delta beta correspond to relative hypermethylation among the cases.

### Comparison to WH dataset

An analysis of differential DNA methylation sites associated with COPD was performed previously by our group using WH subjects in the ICGN. This dataset has been previously described, [15] and additional information of the study design and their baseline characteristics (Additional file 1: Table S2) is provided in Additional file 1. Whole blood samples were assayed for DNA methylation on the Illumina27k array. This dataset included 692 COPD WH cases defined by FEV1/FVC ≤ 0.7 and FEV1 ≤ 80 % predicted, as well as 437 WH controls, with race and ethnicity determined by self-report. Blood draws for cases and controls occurred during routine study office visits. We passed these data through annotation, quality control, probe control, cell type deconvolution, and differential DNA methylation analysis steps that were identical to those used for the PA-SCOPE dataset. We hypothesized that statistical associations for differential methylation of genes between AA and WH may reflect biological differences in disease mechanisms or disease-relevant exposures. We used this ICGN WH dataset to compare differential methylation values to those found in the PA-SCOPE AA cohort. We compared the differential methylation characteristics of COPD-associated CpG sites between AAs and WHs qualitatively in order to identify sites with significant differential methylation in AA without corresponding differential methylation in WH. In order to identify those sites at which the difference in differential methylation was qualitatively greatest between AAs and WHs, we calculated the difference in test statistic (retaining direction of effect) for each CpG site between the PA-SCOPE AA analysis and the ICGN WH analysis.

### Weighted gene comethylation network analysis

WGCNA is a network analysis tool that uses hierarchical clustering of correlated methylation states (transformed using the power adjacency function) between CpG probes to construct weighted comethylation modules [20]. The eigengene of each module mathematically summarizes the comethylation information of all CpGs within each module for modeling purposes, and these eigengenes are subsequently modeled against phenotypic outcomes to show association between phenotypes and comethylation modules. While differential methylation analysis examines one CpG probe at a time, WGCNA incorporates information from all probes in a module to evaluate the module’s association with a trait. Classification of CpG sites and genes within the same COPD-associated module supports coregulation of these genes through DNA methylation. This approach complements the individual CpG site methylation findings by implicating additional genes that may not achieve statistical significance through differential methylation analysis, but may still play a role in phenotypic association through their comethylation within the module.

Weighted gene comethylation networks were constructed using the quality controlled PA-SCOPE dataset as input to the WGCNA R-package by Langfelder and Horvath, [20] and network modules were analyzed for association with COPD in a signed correlation network. Scale-free properties were achieved with a soft thresholding value of 12, resulting in ten modules. Analysis of the eigengenes of significantly COPD-associated modules (measured as the correlation of the gene’s methylation profile with the module eigenvector) was performed to evaluate driver genes for the module (genes whose methylation is most highly correlated to the eigengene methylation). Gene significance is quantified statistically by the Student’s *t*-test statistic for differential methylation between COPD cases and controls, and larger values of gene significance indicate more contribution of the gene’s comethylation pattern to the module’s association with COPD. Results were inspected for genes in the differential methylation top results, as well as for known obstructive lung disease associations from GWAS studies of lung function [65–67] and COPD [68–73]. For each significantly associated eigengene, logistic regression models of COPD case-control status were constructed using the module eigengenes and clinical covariates of age, gender, and pack-years of smoking history to evaluate the robustness of the eigengene association to COPD. COPD-associated WGCNA modules were limited to genes with a stringent module membership (kME value) cutoff of >0.85, as per previously published methods [21]. Gene set enrichment analysis on these limited modules was performed using ConsensusPathDB [74] to compare the COPD-associated module genes to evaluate for enrichment in the Gene Ontology (GO) Biological Processes ontology, Kyoto Encyclopedia of Genes and Genomes (KEGG), and Reactome pathways using hypergeometric testing controlled for multiple testing using the false discovery rate method applied to the number of included genes.

## Additional file

Additional file 1: Supplementary data and methods are provided in Additional file 1. This file includes additional methodological details related to study design, Tables S1 and S2, and Figures S1-S3. (DOCX 3650 kb)

## Abbreviations

AA: Subjects that racially self-identify as African-American
COPD: Chronic obstructive pulmonary disease
CpG: Cytosine phosphate-bond guanine dinucleotide
FEV1: Forced expiratory volume in one second
FVC: Forced vital capacity
GO: Gene ontology
ICGN: International COPD Genetics Network
KEGG: Kyoto Encyclopedia of Genes and Genomes
mQTL: Methylation quantitative trait loci
PA-SCOPE: Pennsylvania Study of Chronic Obstructive Pulmonary Exacerbations
PYH: Pack-year history of smoking
WGCNA: Weighted gene comethylation network analysis
WH: Subjects that racially self-identify as white

## References

[1] Kochanek KD, Murphy SL, Xu J (2015). Deaths: Final Data for 2011. National vital statistics reports : from the Centers for Disease Control and Prevention, National Center for Health Statistics, National Vital Statistics System.

[2] Vestbo J, Hurd SS, Agusti AG, Jones PW, Vogelmeier C, Anzueto A, Barnes PJ, Fabbri LM, Martinez FJ, Nishimura M (2013). Global strategy for the diagnosis, management, and prevention of chronic obstructive pulmonary disease: GOLD executive summary. American journal of respiratory and critical care medicine.

[3] Trends in COPD (Chronic Bronchitis and Emphysema): Morbidity and Mortality [http://www.lung.org/assets/documents/research/copd-trend-report.pdf]. Accessed 6 Mar 2016.

[4] Dransfield MT, Bailey WC (2006). COPD: racial disparities in susceptibility, treatment, and outcomes. Clinics in chest medicine.

[5] Foreman MG, Zhang L, Murphy J, Hansel NN, Make B, Hokanson JE, Washko G, Regan EA, Crapo JD, Silverman EK, DeMeo DL (2011). Early-onset chronic obstructive pulmonary disease is associated with female sex, maternal factors, and African American race in the COPDGene Study. American journal of respiratory and critical care medicine.

[6] State of Lung Disease in Diverse Communities: 2010 [http://action.lung.org/site/DocServer/state-of-lung-disease-in-diverse-communities-2010.pdf]. Accessed 6 Mar 2016.

[7] Han MK, Curran-Everett D, Dransfield MT, Criner GJ, Zhang L, Murphy JR, Hansel NN, DeMeo DL, Hanania NA, Regan EA (2011). Racial differences in quality of life in patients with COPD. Chest.

[8] Manichaikul A, Hoffman EA, Smolonska J, Gao W, Cho MH, Baumhauer H, Budoff M, Austin JH, Washko GR, Carr JJ (2014). Genome-wide study of percent emphysema on computed tomography in the general population. The Multi-Ethnic Study of Atherosclerosis Lung/SNP Health Association Resource Study. American journal of respiratory and critical care medicine.

[9] Sun YV, Smith AK, Conneely KN, Chang Q, Li W, Lazarus A, Smith JA, Almli LM, Binder EB, Klengel T (2013). Epigenomic association analysis identifies smoking-related DNA methylation sites in African Americans. Human genetics.

[10] Fraser HB, Lam LL, Neumann SM, Kobor MS (2012). Population-specificity of human DNA methylation. Genome biology.

[11] Barfield RT, Almli LM, Kilaru V, Smith AK, Mercer KB, Duncan R, Klengel T, Mehta D, Binder EB, Epstein MP (2014). Accounting for population stratification in DNA methylation studies. Genetic epidemiology.

[12] Bjornsson HT, Fallin MD, Feinberg AP (2004). An integrated epigenetic and genetic approach to common human disease. Trends in genetics : TIG.

[13] Breitling LP, Yang R, Korn B, Burwinkel B, Brenner H (2011). Tobacco-smoking-related differential DNA methylation: 27K discovery and replication. American journal of human genetics.

[14] Sakao S, Tatsumi K (2011). The importance of epigenetics in the development of chronic obstructive pulmonary disease. Respirology.

[15] Qiu W, Baccarelli A, Carey VJ, Boutaoui N, Bacherman H, Klanderman B, Rennard S, Agusti A, Anderson W, Lomas DA, DeMeo DL (2012). Variable DNA methylation is associated with chronic obstructive pulmonary disease and lung function. American journal of respiratory and critical care medicine.

[16] Wan ES, Qiu W, Baccarelli A, Carey VJ, Bacherman H, Rennard SI, Agusti A, Anderson W, Lomas DA, Demeo DL (2012). Cigarette smoking behaviors and time since quitting are associated with differential DNA methylation across the human genome. Human molecular genetics.

[17] Zeilinger S, Kuhnel B, Klopp N, Baurecht H, Kleinschmidt A, Gieger C, Weidinger S, Lattka E, Adamski J, Peters A (2013). Tobacco smoking leads to extensive genome-wide changes in DNA methylation. PloS one.

[18] Saccone NL, Wang JC, Breslau N, Johnson EO, Hatsukami D, Saccone SF, Grucza RA, Sun L, Duan W, Budde J (2009). The CHRNA5-CHRNA3-CHRNB4 nicotinic receptor subunit gene cluster affects risk for nicotine dependence in African-Americans and in European-Americans. Cancer research.

[19] Zhu G, Warren L, Aponte J, Gulsvik A, Bakke P, Anderson WH, Lomas DA, Silverman EK, Pillai SG (2007). The SERPINE2 gene is associated with chronic obstructive pulmonary disease in two large populations. American journal of respiratory and critical care medicine.

[20] Langfelder P, Horvath S (2008). WGCNA: an R package for weighted correlation network analysis. BMC bioinformatics.

[21] Rickabaugh TM, Baxter RM, Sehl M, Sinsheimer JS, Hultin PM, Hultin LE, Quach A, Martinez-Maza O, Horvath S, Vilain E, Jamieson BD (2015). Acceleration of age-associated methylation patterns in HIV-1-infected adults. PloS one.

[22] Wan ES, Qiu W, Baccarelli A, Carey VJ, Bacherman H, Rennard SI, Agusti A, Anderson WH, Lomas DA, DeMeo DL (2012). Systemic steroid exposure is associated with differential methylation in chronic obstructive pulmonary disease. American journal of respiratory and critical care medicine.

[23] GTEx Consortium. The Genotype-Tissue Expression (GTEx) pilot analysis: multitissue gene regulation in humans. Science. 2015;348:648–60. 10.1126/science.1262110 PMC4547484 25954001

[24] Wu X, Sun X, Chen C, Bai C, Wang X (2014). Dynamic gene expressions of peripheral blood mononuclear cells in patients with acute exacerbation of chronic obstructive pulmonary disease: a preliminary study. Critical care.

[25] Wijkstrom-Frei C, El-Chemaly S, Ali-Rachedi R, Gerson C, Cobas MA, Forteza R, Salathe M, Conner GE (2003). Lactoperoxidase and human airway host defense. American journal of respiratory cell and molecular biology.

[26] Jin N, Kolliputi N, Gou D, Weng T, Liu L (2006). A novel function of ionotropic gamma-aminobutyric acid receptors involving alveolar fluid homeostasis. The Journal of biological chemistry.

[27] Rowan-Carroll A, Halappanavar S, Williams A, Somers CM, Yauk CL (2013). Mice exposed in situ to urban air pollution exhibit pulmonary alterations in gene expression in the lipid droplet synthesis pathways. Environmental and molecular mutagenesis.

[28] Igartua C, Myers RA, Mathias RA, Pino-Yanes M, Eng C, Graves PE, Levin AM, Del-Rio-Navarro BE, Jackson DJ, Livne OE (2015). Ethnic-specific associations of rare and low-frequency DNA sequence variants with asthma. Nature communications.

[29] TCNG: The Cancer Network Galaxy [http://tcng.hgc.jp/index.html]. Accessed 6 Mar 2016.

[30] Baughman RP, Culver DA (2015). Sarcoidosis. Clinics in chest medicine.

[31] Kanehisa M, Goto S (2000). KEGG: kyoto encyclopedia of genes and genomes. Nucleic acids research.

[32] Kumar A, Das S, Agrawal A, Mukhopadhyay I, Ghosh B (2015). Genetic association of key Th1/Th2 pathway candidate genes, IRF2, IL6, IFNGR2, STAT4 and IL4RA, with atopic asthma in the Indian population. Journal of human genetics.

[33] Li X, Zhang X, Leathers R, Makino A, Huang C, Parsa P, Macias J, Yuan JX, Jamieson SW, Thistlethwaite PA (2009). Notch3 signaling promotes the development of pulmonary arterial hypertension. Nature medicine.

[34] Wei C, Qiu J, Zhou Y, Xue Y, Hu J, Ouyang K, Banerjee I, Zhang C, Chen B, Li H, et al. Repression of the Central Splicing Regulator RBFox2 Is Functionally Linked to Pressure Overload-Induced Heart Failure. Cell Rep. 2015. doi:10.1016/j.celrep.2015.02.013. [Epub ahead of print]. 10.1016/j.celrep.2015.02.013 PMC4559494 25753418

[35] Smith AK, Kilaru V, Kocak M, Almli LM, Mercer KB, Ressler KJ, Tylavsky FA, Conneely KN (2014). Methylation quantitative trait loci (meQTLs) are consistently detected across ancestry, developmental stage, and tissue type. BMC genomics.

[36] Carrell RW, Jeppsson JO, Laurell CB, Brennan SO, Owen MC, Vaughan L, Boswell DR (1982). Structure and variation of human alpha 1-antitrypsin. Nature.

[37] Bhat TA, Panzica L, Kalathil SG, Thanavala Y (2015). Immune Dysfunction in Patients with Chronic Obstructive Pulmonary Disease. Annals of the American Thoracic Society.

[38] Retamales I, Elliott WM, Meshi B, Coxson HO, Pare PD, Sciurba FC, Rogers RM, Hayashi S, Hogg JC (2001). Amplification of inflammation in emphysema and its association with latent adenoviral infection. American journal of respiratory and critical care medicine.

[39] Lommatzsch M, Bratke K, Knappe T, Bier A, Dreschler K, Kuepper M, Stoll P, Julius P, Virchow JC (2010). Acute effects of tobacco smoke on human airway dendritic cells in vivo. The European respiratory journal.

[40] Hoenderdos K, Condliffe A (2013). The neutrophil in chronic obstructive pulmonary disease. American journal of respiratory cell and molecular biology.

[41] Brightling CE, Monteiro W, Ward R, Parker D, Morgan MD, Wardlaw AJ, Pavord ID (2000). Sputum eosinophilia and short-term response to prednisolone in chronic obstructive pulmonary disease: a randomised controlled trial. Lancet.

[42] Brightling CE, McKenna S, Hargadon B, Birring S, Green R, Siva R, Berry M, Parker D, Monteiro W, Pavord ID, Bradding P (2005). Sputum eosinophilia and the short term response to inhaled mometasone in chronic obstructive pulmonary disease. Thorax.

[43] Culley FJ (2009). Natural killer cells in infection and inflammation of the lung. Immunology.

[44] Wedzicha JA (2015). Mechanisms of Chronic Obstructive Pulmonary Disease Exacerbations. Annals of the American Thoracic Society.

[45] Papi A, Bellettato CM, Braccioni F, Romagnoli M, Casolari P, Caramori G, Fabbri LM, Johnston SL (2006). Infections and airway inflammation in chronic obstructive pulmonary disease severe exacerbations. American journal of respiratory and critical care medicine.

[46] Guida F, Sandanger TM, Castagne R, Campanella G, Polidoro S, Palli D, Krogh V, Tumino R, Sacerdote C, Panico S (2015). Dynamics of smoking-induced genome-wide methylation changes with time since smoking cessation. Human molecular genetics.

[47] Van Schayck CP, Loozen JM, Wagena E, Akkermans RP, Wesseling GJ (2002). Detecting patients at a high risk of developing chronic obstructive pulmonary disease in general practice: cross sectional case finding study. BMJ.

[48] Takahashi T, Ichinose M, Inoue H, Shirato K, Hattori T, Takishima T (2003). Underdiagnosis and undertreatment of COPD in primary care settings. Respirology.

[49] Lange P, Celli B, Agusti A, Boje Jensen G, Divo M, Faner R, Guerra S, Marott JL, Martinez FD, Martinez-Camblor P (2015). Lung-Function Trajectories Leading to Chronic Obstructive Pulmonary Disease. The New England journal of medicine.

[50] Bibikova M, Le J, Barnes B, Saedinia-Melnyk S, Zhou L, Shen R, Gunderson KL (2009). Genome-wide DNA methylation profiling using Infinium(R) assay. Epigenomics.

[51] Kotton DN, Morrisey EE (2014). Lung regeneration: mechanisms, applications and emerging stem cell populations. Nature medicine.

[52] Gao X, Jia M, Zhang Y, Breitling LP, Brenner H (2015). DNA methylation changes of whole blood cells in response to active smoking exposure in adults: a systematic review of DNA methylation studies. Clinical epigenetics.

[53] Li L, Choi JY, Lee KM, Sung H, Park SK, Oze I, Pan KF, You WC, Chen YX, Fang JY (2012). DNA methylation in peripheral blood: a potential biomarker for cancer molecular epidemiology. Journal of epidemiology/Japan Epidemiological Association.

[54] Anthonisen NR, Manfreda J, Warren CP, Hershfield ES, Harding GK, Nelson NA (1987). Antibiotic therapy in exacerbations of chronic obstructive pulmonary disease. Annals of internal medicine.

[55] Du P, Zhang X, Huang CC, Jafari N, Kibbe WA, Hou L, Lin SM (2010). Comparison of Beta-value and M-value methods for quantifying methylation levels by microarray analysis. BMC bioinformatics.

[56] R Core Team. R: A language and environment for statistical computing. Vienna: R Foundation for StatisticalComputing; 2016. https://www.R-project.org/.

[57] Huber W, Carey VJ, Gentleman R, Anders S, Carlson M, Carvalho BS, Bravo HC, Davis S, Gatto L, Girke T (2015). Orchestrating high-throughput genomic analysis with Bioconductor. Nature methods.

[58] Pan Du SD, Sven Bilke, Tim Triche, Jr., Moiz Bootwalla: methylumi: Handle Illumina methylation data. 2.16 edition; 2014.

[59] Lawrence M, Huber W, Pages H, Aboyoun P, Carlson M, Gentleman R, Morgan MT, Carey VJ (2013). Software for computing and annotating genomic ranges. PLoS computational biology.

[60] Pidsley R, CC YW, Volta M, Lunnon K, Mill J, Schalkwyk LC (2013). A data-driven approach to preprocessing Illumina 450K methylation array data. BMC genomics.

[61] Chen YA, Choufani S, Ferreira JC, Grafodatskaya D, Butcher DT, Weksberg R (2011). Sequence overlap between autosomal and sex-linked probes on the Illumina HumanMethylation27 microarray. Genomics.

[62] Bock C, Tomazou EM, Brinkman AB, Muller F, Simmer F, Gu H, Jager N, Gnirke A, Stunnenberg HG, Meissner A (2010). Quantitative comparison of genome-wide DNA methylation mapping technologies. Nature biotechnology.

[63] Houseman EA, Accomando WP, Koestler DC, Christensen BC, Marsit CJ, Nelson HH, Wiencke JK, Kelsey KT (2012). DNA methylation arrays as surrogate measures of cell mixture distribution. BMC bioinformatics.

[64] Ritchie ME, Phipson B, Wu D, Hu Y, Law CW, Shi W, Smyth GK (2015). limma powers differential expression analyses for RNA-sequencing and microarray studies. Nucleic acids research.

[65] Repapi E, Sayers I, Wain LV, Burton PR, Johnson T, Obeidat M, Zhao JH, Ramasamy A, Zhai G, Vitart V (2010). Genome-wide association study identifies five loci associated with lung function. Nature genetics.

[66] Soler Artigas M, Loth DW, Wain LV, Gharib SA, Obeidat M, Tang W, Zhai G, Zhao JH, Smith AV, Huffman JE (2011). Genome-wide association and large-scale follow up identifies 16 new loci influencing lung function. Nature genetics.

[67] Hancock DB, Eijgelsheim M, Wilk JB, Gharib SA, Loehr LR, Marciante KD, Franceschini N, van Durme YM, Chen TH, Barr RG (2010). Meta-analyses of genome-wide association studies identify multiple loci associated with pulmonary function. Nature genetics.

[68] Pillai SG, Ge D, Zhu G, Kong X, Shianna KV, Need AC, Feng S, Hersh CP, Bakke P, Gulsvik A (2009). A genome-wide association study in chronic obstructive pulmonary disease (COPD): identification of two major susceptibility loci. PLoS genetics.

[69] Celedon JC, Lange C, Raby BA, Litonjua AA, Palmer LJ, DeMeo DL, Reilly JJ, Kwiatkowski DJ, Chapman HA, Laird N (2004). The transforming growth factor-beta1 (TGFB1) gene is associated with chronic obstructive pulmonary disease (COPD). Human molecular genetics.

[70] DeMeo DL, Mariani T, Bhattacharya S, Srisuma S, Lange C, Litonjua A, Bueno R, Pillai SG, Lomas DA, Sparrow D (2009). Integration of genomic and genetic approaches implicates IREB2 as a COPD susceptibility gene. American journal of human genetics.

[71] Cho MH, Boutaoui N, Klanderman BJ, Sylvia JS, Ziniti JP, Hersh CP, DeMeo DL, Hunninghake GM, Litonjua AA, Sparrow D (2010). Variants in FAM13A are associated with chronic obstructive pulmonary disease. Nature genetics.

[72] Cho MH, Castaldi PJ, Wan ES, Siedlinski M, Hersh CP, Demeo DL, Himes BE, Sylvia JS, Klanderman BJ, Ziniti JP (2012). A genome-wide association study of COPD identifies a susceptibility locus on chromosome 19q13. Human molecular genetics.

[73] Cho MH, McDonald ML, Zhou X, Mattheisen M, Castaldi PJ, Hersh CP, Demeo DL, Sylvia JS, Ziniti J, Laird NM (2014). Risk loci for chronic obstructive pulmonary disease: a genome-wide association study and meta-analysis. The Lancet Respiratory medicine.

[74] Kamburov A, Pentchev K, Galicka H, Wierling C, Lehrach H, Herwig R (2011). ConsensusPathDB: toward a more complete picture of cell biology. Nucleic acids research.

