# Supplementary material for: Differential DNA methylation marks and gene comethylation of COPD in African-Americans with COPD exacerbations
Source: Respir Res. 2016 Nov 5;17:143. doi: 10.1186/s12931-016-0459-8 (PMC5097392; doi:10.1186/s12931-016-0459-8)
Supplement: Additional file 1: — Supplementary data and methods are provided in Additional file 1. This file includes additional methodological details related to study design, Tables S1 and S2, and Figures S1-S3. (DOCX 3650 kb) [file 12931_2016_459_MOESM1_ESM.docx]

**Additional File 1: Supplemental Methods and Data**

**Title: Differential DNA Methylation Marks and Gene Comethylation of COPD in African-Americans with COPD Exacerbations**

**Authors:** Robert Busch, MD^1^, Weiliang Qiu, PhD^1^, Jessica Lasky-Su, PhD^1^, Jarrett Morrow, PhD^1^, Gerard Criner, MD^2^, and Dawn DeMeo, MD MPH^1^

**Affiliations:** ^1^Channing Division of Network Medicine, Department of Medicine, Brigham and Women's Hospital, Boston, MA/USA; ^2^Temple Lung Center, Temple University Health System, Philadelphia, PA/USA

**Supplemental Methods:**

Participants in the PA-SCOPE study met the following inclusion criteria: male or female subjects between 40-80 years of age, self-identified as African-American (AA) or non-Hispanic White, current or former smokers, with a history of cigarette smoking of >20 pack-years. Subjects had to be willing and able to understand study procedures, undergo study procedures, and provide informed consent for the study. Exclusion criteria for cases and controls included an inability or unwillingness to complete required procedures or visits, inability or unwillingness to provide informed consent, a personal history of asthma or any other significant respiratory disease (specifically assessed for history of pulmonary fibrosis, bronchiectasis, mediastinal mass or adenopathy, or a pulmonary mass), a life expectancy < 6 months due to any cause, a smoking history of <20 pack-years, an inability to read or write English, or a history of alpha-1 antitrypsin deficiency per self-report and medical history.

The PA-SCOPE COPD case group met the following additional inclusion criteria: a history of airway obstruction and spirometric evidence of COPD by FEV1 < 70% and FEV1/FVC <0.7 of predicted normals; COPD exacerbation was the primary cause for the inpatient hospitalization as determined by the patient's care team; documentation of hyperinflation on the chest x-ray was required to be present. Exclusion criteria specific to the COPD case group included a lack of documentation of COPD (on chest x-ray, spirometry, or physical exam), lack of obstructive deficit (defined as FEV1/FVC >0.7) on pulmonary function testing, substantial bronchodilatory response/reversibility in the obstructive deficit on spirometry (increase in FEV1 >15% on prior pulmonary function testing), or if the inpatient hospitalization was believed to have been precipitated by causes other than a COPD exacerbation.

The PA-SCOPE control group subjects met the study inclusion criteria and did not have a personal history of COPD. Additional exclusion criteria for the PA-SCOPE control group included, an FEV1 <70% of predicted normal or a FEV1/FVC ratio <0.7 (i.e. objective evidence of a spirometric diagnosis of airways obstruction, significant lung function limitation, or COPD).

The PA-SCOPE non-Hispanic white population was limited to <50 participants, which did not give us adequate power to detect associations, so comparisons between racial groups were conducted in the International COPD Genetics Network study dataset. White subjects (WH) examined in our investigation were selected from the multi-center International COPD Genetics Network (ICGN) study dataset. ICGN recruited COPD probands and siblings in a family-based study design. All subjects had >5 pack-years of smoking history. Probands were 45-65 years of age and had a diagnosis of COPD based on airflow limitation, with a post-bronchodilator FEV1 < 60% predicted and FEV1/Vital Capacity (VC) 90% predicted. Subjects with alpha-1 antitrypsin deficiency were excluded. To be eligible for study inclusion, an eligible sibling with >5 pack-years of smoking history was required. In total, 1910 white individuals from 635 families were included in the original study dataset.

For our investigation, we reclassified COPD case subjects in ICGN using a definition of COPD consistent with that used in PA-SCOPE (FEV1/FVC <0.7 and FEV1 <80% of predicted). Subjects not meeting these spirometric criteria were used as controls.

**Supplemental Tables:**

| **Chromosome** | **CpG Probe** | **Nearest Gene** | **PA-SCOPE Association p-value** | **PA-SCOPE Association t-value** | **ICGN Association t-value** | **Test-statistic Difference** | |
| --- | --- | --- | --- | --- | --- | --- | --- |
| 5 | cg16361890** | *MAML1* | 8.19E-08 | -5.48 | -0.58 | -4.90 | |
| 22 | cg00615377** | *RBFOX2* | 1.20E-07 | -5.40 | -1.16 | -4.24 | |
| 9 | cg12971694** | *CD72* | 2.05E-06 | -4.83 | -1.25 | -3.58 | |
| 12 | cg22566906** | *GRASP* | 7.27E-06 | -4.55 | -0.97 | -3.59 | |
| 4 | cg02635407 | *SH3TC1* | 7.47E-06 | -4.55 | -3.21 | -1.34 | |
| 11 | cg25634666 | *FOLR3* | 3.23E-05 | -4.21 | -2.23 | -1.98 | |
| 10 | cg18390025 | *ELOVL3* | 3.40E-05 | -4.20 | -1.95 | -2.24 | |
| 5 | cg10257049** | *FAXDC2* | 3.58E-05 | -4.19 | -0.43 | -3.76 | |
| 19 | cg27461196 | *FXYD1/ LGI4* | 4.27E-05 | -4.14 | -3.61 | -0.53 | |
| 6 | cg00333528 | *GABRR1* | 4.43E-05 | -4.14 | -1.15 | -2.98 | |
| 21 | cg17356733** | *IFNGR2* | 4.56E-05 | -4.13 | -0.69 | -3.44 | |
| 17 | cg24489015** | *LPO* | 5.82E-05 | -4.07 | 0.28 | -4.35 | |
| **Supplemental Table 1: Comparison to WH Dataset.** For each CpG site, test statistics for association with COPD were calculated from linear mixed models adjusted for age, gender, pack years of smoking, assay batch, and cell type in the PA-SCOPE (AA) and ICGN (WH) datasets. The difference in test statistic between PA-SCOPE and ICGN was approximately normally distributed over the 19302 probes analyzed. Two-sided 95% confidence intervals were computed for the distribution, with the 97.5% confidence boundary corresponding to a test-statistic difference of 2.97 and the 2.5% confidence boundary corresponding to a test-statistic difference of -3.43. The test-statistic difference values of 7 of the 12 top differentially methylated CpG sites associated with COPD in the PA-SCOPE dataset were in the lower tail of this distribution, meaning they were statistically differentially methylated in the PA-SCOPE dataset and not in the ICGN dataset. | | | | | | |  |

|  | **Cases** | | **Controls** | | | **p-value** | |
| --- | --- | --- | --- | --- | --- | --- | --- |
| N= | 678 |  | 427 |  | |  | |
| Age (years) | 59.20 | ± 7.4 | 54.90 | ± 9.0 | | 4.8E-16 | |
| FEV1/FVC Ratio | 0.42 | ± 0.136 | 0.77 | ± 0.043 | | 6.3E-318 | |
| FEV1 percent predicted | 45.36 | ± 17.8 | 104.48 | ± 15.8 | | 2.9E-317 | |
| FVC percent predicted | 80.08 | ± 20.6 | 108.71 | ± 16.7 | | 7.6E-110 | |
| Gender (% male) | 389 | (57.4) | 216 | (50.6) | | 0.03 | |
| Pack-Years of Smoking | 49.71 | ± 26.0 | 29.44 | ± 20.7 | | 5.0E-226 | |
| **Supplemental Table 2. Baseline Statistics Among Whites in ICGN.** Data is presented as | | | | | | | |
| count with proportion in parentheses or mean with standard deviation. Student's | | | | | | | |
| t-test was used to calculate the p-value for difference in means between cases and | | | | | | | |
| controls for quantitative variables, while a chi-square test was used to calculate the | | | | | | | |
| difference in proportion for the categorical variable (gender). | | | | |  | |  |

**Supplemental Figures:**


**Supplemental Figure 1: Comparison to WH Dataset.** For each CpG site, test statistics were compared

between the PA-SCOPE and ICGN datasets. The difference in test statistic was normally distributed over

the 19302 probes analyzed. Notably, many of the CpG sites found to be differentially methy-

lated in association with COPD in PA-SCOPE were in the lower tail of this distribution.

**Supplemental Figure 2: Network Topology Heatmap.** Heatmap representation of the topological overlap-based dissimilarity matrix calculated from PA-SCOPE weighted gene comethylation network analysis. Hierarchical clustering was used to construct clustering dendrograms to separate CpG sites into modules based on comethylation correlation. Module assignment colors based on clustering are represented along the x- and y-axis.

**Supplemental Figure 3: Differential Methylation Boxplots.** Boxplots of the beta value (absolute methylation ranging from 0 to 1, corresponding to 0 to 100% methylated) for each of the twelve statistically significantly differentially methylated CpG sites reported in the Results section. These boxplots do not account for covariate adjustment. Boxplots are shown for COPD cases and controls in both PA-SCOPE and ICGN. Red asterisks represent the mean absolute beta for the samples in each group.
